# Management and Therapeutic Considerations of Hydrogen Peroxide-Induced Necrotizing Gastroenteritis: A Case Report

**DOI:** 10.7759/cureus.96224

**Published:** 2025-11-06

**Authors:** Olivia Mesdjian, Marral K Pourmoghadam, Dolores Ivkovic, Maryann H Saba, David Hernandez, Frederick Tiesenga

**Affiliations:** 1 School of Medicine, St. George's University School of Medicine, St. George's, GRD; 2 Surgery, St. George's University School of Medicine, St. George's, GRD; 3 General Surgery, West Suburban Medical Center, Chicago, USA

**Keywords:** caustic injury, conservative management, endoscopy, gastrointestinal necrosis, hydrogen peroxide ingestion, hyperbaric oxygen therapy, necrotizing gastroenteritis, portal venous gas, roux-en-y

## Abstract

Necrotizing gastroenteritis is a rare but severe condition characterized by extensive inflammation and mucosal necrosis of the gastrointestinal tract. It is most commonly seen in neonates, infants, and immunocompromised individuals. We present a rare case of a 46-year-old female patient who developed necrotizing gastroenteritis following accidental ingestion of 3% hydrogen peroxide, manifesting as three days of epigastric pain, heartburn, and recurrent emesis. This report details the patient’s clinical presentation, diagnostic evaluation, therapeutic strategies, and management course. The case emphasizes the importance of clinical suspicion, timely intervention, and consideration of both surgical and conservative treatment approaches, with particular attention to long-term functional complications and quality of life.

## Introduction

Hydrogen peroxide is a widely used oxidizing agent in homes and industry. Household solutions (~3%) are generally safe when used as directed, but misuse can be harmful; high-concentration industrial formulations (up to ~70%) are caustic and have been linked to severe injury and death [1]. Hydrogen peroxide causes damage via three mechanisms: direct caustic injury, oxygen gas formation, and lipid peroxidation, with even 3% household solutions associated with portal venous gas, pneumatosis, and upper gastrointestinal bleeding [2]. 

Early esophagogastroduodenoscopy (EGD), within 12-24 hours, is recommended for risk stratification using systems such as Zargar Classification of caustic esophageal injury, whereas endoscopy is generally postponed during the friable healing of approximately 5-15 days [3]. Zargar's classification is the most commonly used endoscopic scale to assess esophageal damage from caustic ingestion [4]. Classification is accomplished with a Grade of 0 to 4. Grade 0 presents with normal findings, while Grade 1 shows mucosal edema and hyperemia. Grades 2 and 3 are both subdivided as follows: 2A is characterized by superficial injury such as ulcerations, erosions, and exudates, 2B is denoted by deeper ulcerations, 3A indicates focal necrosis, and 3B indicates extensive necrosis. Lastly, Grade 4 indicates perforation [4].

It is important to distinguish between benign gastric emphysema and emphysematous (necrotizing) gastritis, which has been linked to over 50% mortality in some reports, with patients receiving more aggressive treatment, including prompt resuscitation, broad-spectrum antibiotics, and early surgical consultation [5]. In patients with prior bariatric surgery, anastomoses and rerouted flow dynamics may change how injury manifests and complicate interpretation, supporting the need for tailored imaging strategies in this population of patients [6]. 

This report highlights the case of a middle-aged female with prior Roux-en-Y gastric bypass who developed necrotizing gastroenteritis after accidentally ingesting 3% hydrogen peroxide, presenting with acute epigastric pain accompanied by hematemesis. We outline the patient’s clinical course, including the diagnostic work-up and successful nonoperative management.

## Case presentation

A 46-year-old Hispanic woman with a past surgical history of cholecystectomy and Roux-en-Y gastric bypass (six years ago) presented to the emergency department with worsening abdominal pain. After several days of heartburn and non-bilious vomiting, the pain intensified after inadvertently ingesting a “gulp” of 3% hydrogen peroxide, which she mistook for water while half asleep. Following ingestion, she experienced multiple episodes of hematemesis accompanied by abdominal distention, increased pyrosis, and paresthesia. She denied chest pain, dyspnea, fever, chills, jaundice, diarrhea, or new systemic complaints.

Her history was notable for recurrent abdominal pain with associated nausea and emesis, for which she had undergone several prior endoscopies. The most recent esophagogastroduodenoscopy, two years prior to presentation, revealed Los Angeles grade A reflux esophagitis, findings suggestive of short-segment Barrett’s esophagus (biopsies negative for intestinal metaplasia), a 3-cm hiatal hernia, and postsurgical anatomy consistent with Roux-en-Y gastric bypass. Gastric biopsies were negative for *Helicobacter pylori*. Additional medical conditions included hypertension, asthma, anemia, pancreatitis, uterine fibroids, and depression, which were deemed noncontributory to the current presentation. She reported daily cigarette smoking and occasional alcohol use. Her only drug allergy was codeine, which caused dyspnea.

On examination, she appeared well nourished and fully oriented. Vital signs were within normal limits except for elevated blood pressure (182/74 mmHg). Abdominal examination revealed a soft, nondistended abdomen with normoactive bowel sounds and moderate epigastric tenderness without masses. The remainder of her physical exam was unremarkable.

Laboratory evaluation on admission revealed significant leukocytosis with elevated absolute neutrophil and lymphocyte count, mild microcytic anemia, and elevated red cell distribution width. Comprehensive metabolic panel, liver function tests, urinalysis, and lactate were within normal limits (Table 1).

**Table 1:** Laboratory test results on admission BUN: blood urea nitrogen

Comprehensive Metabolic Panel	Result	Reference range
Glucose	109 mg/dL	70-99 mg/dL
Blood urea nitrogen	8 mg/dL	7-25 mg/dL
Creatinine	0.74 mg/dL	0.6-1.3 mg/dL
BUN/Creatinine ratio	11	6-20
Sodium	136 mmol/L	133-144 mmol/L
Potassium	3.8 mmol/L	3.5-5.1 mmol/L
Chloride	102 mmol/L	98-109 mmol/L
Carbon dioxide	25 mmol/L	21-31 mmol/L
Calcium	8.9 mg/dL	8.6-10.3 mg/dL
Liver Function Test	Result	Reference range
Aspartate aminotransferase	22 U/L	10-40 U/L
Alanine aminotransferase	18 U/L	7-56 U/L
Alkaline phosphatase	48 U/L	35-104 U/L
Total bilirubin	0.7 mg/dL	0.0-1.0 mg/dL
Lactate Test	Result	Reference range
Venous lactate	1.0 mmol/L	0.2-2.2 mmol/L
Complete Blood Count	Result	Reference
White blood cell	20.0 x 10³/μl	4.0-11 x 10³/μl
Red blood cell	4.56 x 10^6^/μl	3.63-5.04 x 10^6^/μl
Hemoglobin	11.8 g/dL	12.0-15.3 g/dL
Hematocrit	36.3%	34.7-45.1%
Mean corpuscular volume	79.8 fL	80-100 fL
Mean corpuscular hemoglobin	25.9 pg	26-34 pg
Mean corpuscular hemoglobin concentration	32.4 g/dL	32.5-35.8 g/dL
Red cell distribution width	18.9%	11.9-15.9 %
Platelets	203 x 10³/μl	150-450 10³/μl
Mean platelet volume	9.4 fL	6.8-10.2 fL
Complete Blood Count Differential	Result	Reference range
Neutrophils	56.2%	40-70 %
Lymphocytes	36.9%	20-40 %
Monocytes	6.1%	2-8 %
Eosinophils	0.5%	1-4%
Basophils	0.3%	0-1%
Neutrophils absolute	10.9 x 10³/μl	1.8-7.5 x 10³/μl
Lymphocytes absolute	7.0 x 10³/μl	1.0-4.0 x 10³/μl
Monocytes absolute	0.8 x 10³/μl	0.2-0.8 x 10³/μl
Eosinophils absolute	0.1 x 10³/μl	0.0-0.5 x 10³/μl
Basophils absolute	0.1 x 10³/μl	0.0-0.1 x 10³/μl
Urinalysis	Result	Reference range
Color	Yellow	Yellow, straw colored
Appearance	Hazy	Clear
Ketones	40	Negative
Blood	Trace	Negative

**Figure 1:**
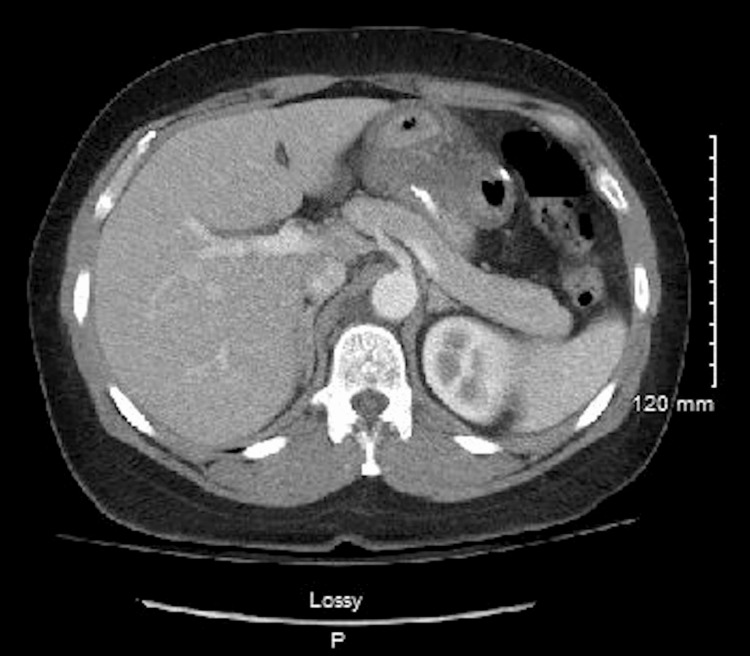
Axial contrast-enhanced CT image of the upper abdomen CT image demonstrates the Roux-en-Y anastomosis region, with circumferential wall thickening and submucosal edema involving the distal stomach and proximal jejunal loops, producing a “halo” appearance.

A contrast-enhanced computed tomography (CT) scan of the abdomen and pelvis showed findings consistent with nonspecific gastroenteritis, with marked circumferential edema and wall thickening of the distal stomach and jejunal loops, including at the Roux-en-Y anastomosis (Figure 1).

Differential diagnoses at this stage included necrotizing gastroenteritis, gastritis, and small bowel obstruction (Table 2).

**Table 2:** Comparative clinical features of necrotizing gastroenteritis and associated differential diagnoses While conditions may share symptoms such as abdominal pain and emesis, key differences in presentation and diagnostic findings help distinguish necrotizing gastroenteritis [11].

Condition	Typical Presentation	Similarity to Necrotizing Gastroenteritis	Key Differences from Necrotizing Gastroenteritis
Necrotizing Gastroenteritis	Sudden onset of severe abdominal pain, emesis (often hematemesis), diarrhea (may be bloody), fever, signs of sepsis or peritonitis in advanced cases [7].		CT: Bowel wall thickening, pneumatosis intestinalis, portal venous gas, free fluid, or gas if perforation. Endoscopy: Edematous, friable, necrotic mucosa, ulcerations, possibly bleeding [8].
Gastritis	Epigastric pain or discomfort, nausea, vomiting, bloating, anorexia; may have melena if bleeding occurs; often subacute or chronic in onset [9].	Both can present with emesis and abdominal pain. CT can show mild gastric wall thickening.	Gastritis typically lacks signs of systemic toxicity, bloody diarrhea, or peritonitis; it's usually more chronic and localized to the stomach. CT: Often normal or shows mild gastric wall thickening. Endoscopy: Erythema, erosions, mucosal edema, sometimes petechiae or superficial ulceration, confined to the stomach only [9].
Small Bowel Obstruction (SBO)	Crampy abdominal pain, nausea, vomiting (may be feculent in distal obstruction), abdominal distension, obstipation, high-pitched bowel sounds early on [10].	Both can present with emesis and abdominal pain	SBO usually features signs of mechanical obstruction (distension, no flatus/stool), not diarrhea; no mucosal necrosis or systemic sepsis unless complicated by ischemia or perforation. CT: Dilated loops of small bowel proximal to obstruction, transition point, collapsed distal bowel; may show signs of ischemia (stranding, decreased wall enhancement) if severe [10].

Given the patient’s prior gastric bypass and marked leukocytosis, she was admitted for inpatient management and anticipated upper endoscopy. Treatment was initiated with intravenous fluids, pantoprazole, piperacillin-tazobactam, ondansetron, famotidine, aluminum hydroxide, and bowel rest.

**Figure 2:**
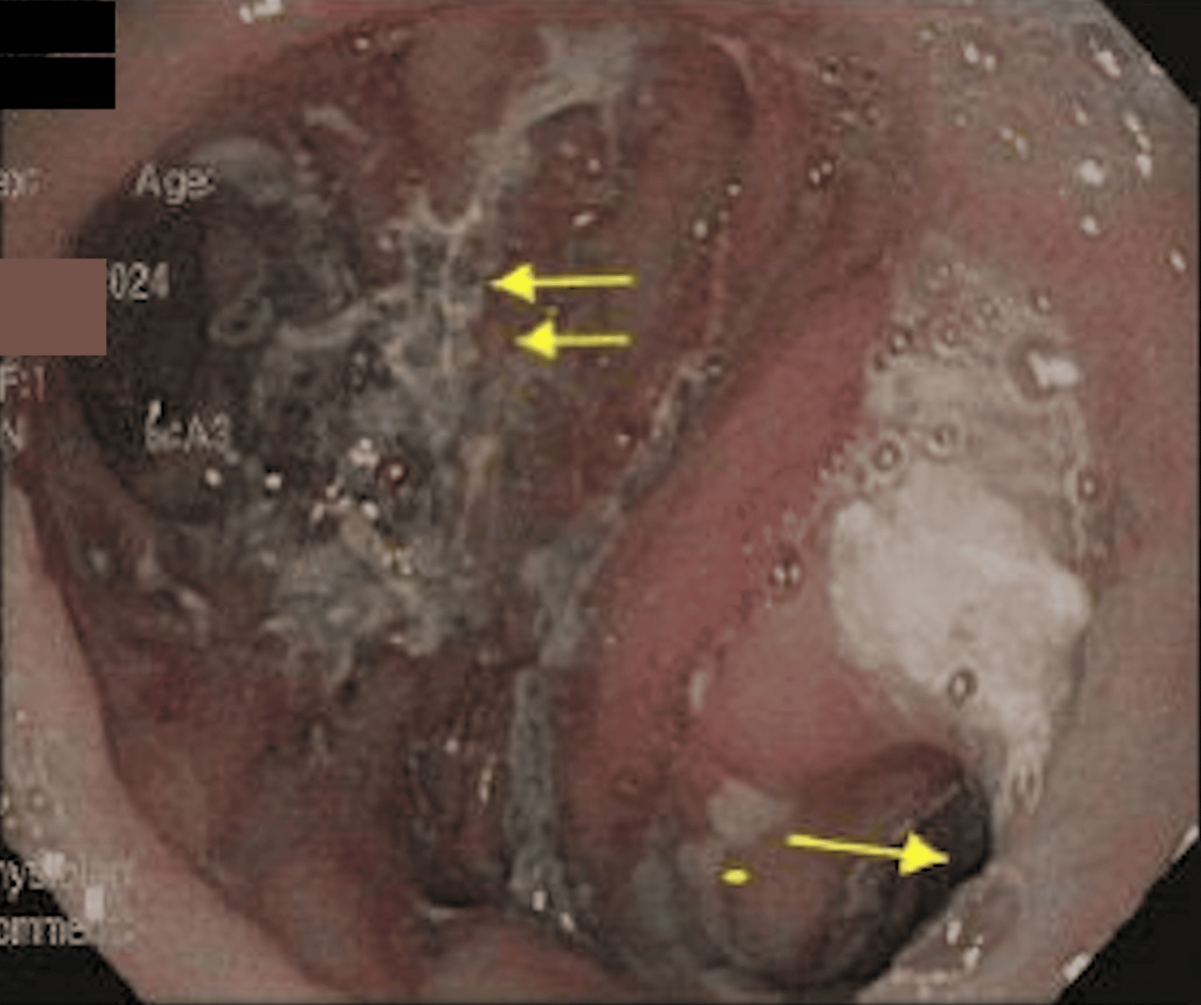
Endoscopic image of the gastric body Evidence of a prior Roux-en-Y gastric bypass surgery is seen.The single arrow marks the jejunal loops. The double arrows highlight mucosal necrosis with surrounding congestion and edema.

**Figure 3:**
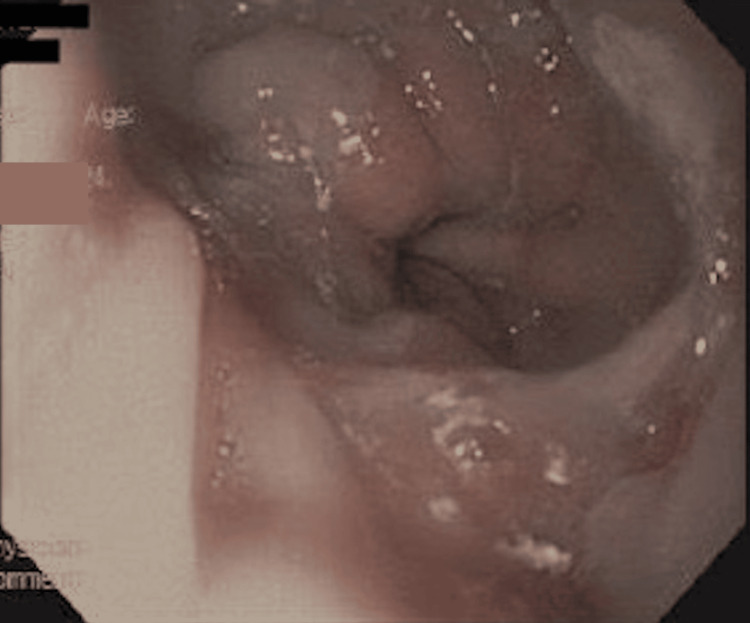
Endoscopic image of the gastroesophageal junction Findings consistent with LA grade A esophagitis: tiny, non-confluent mucosal breaks without active bleeding [12].

**Figure 4:**
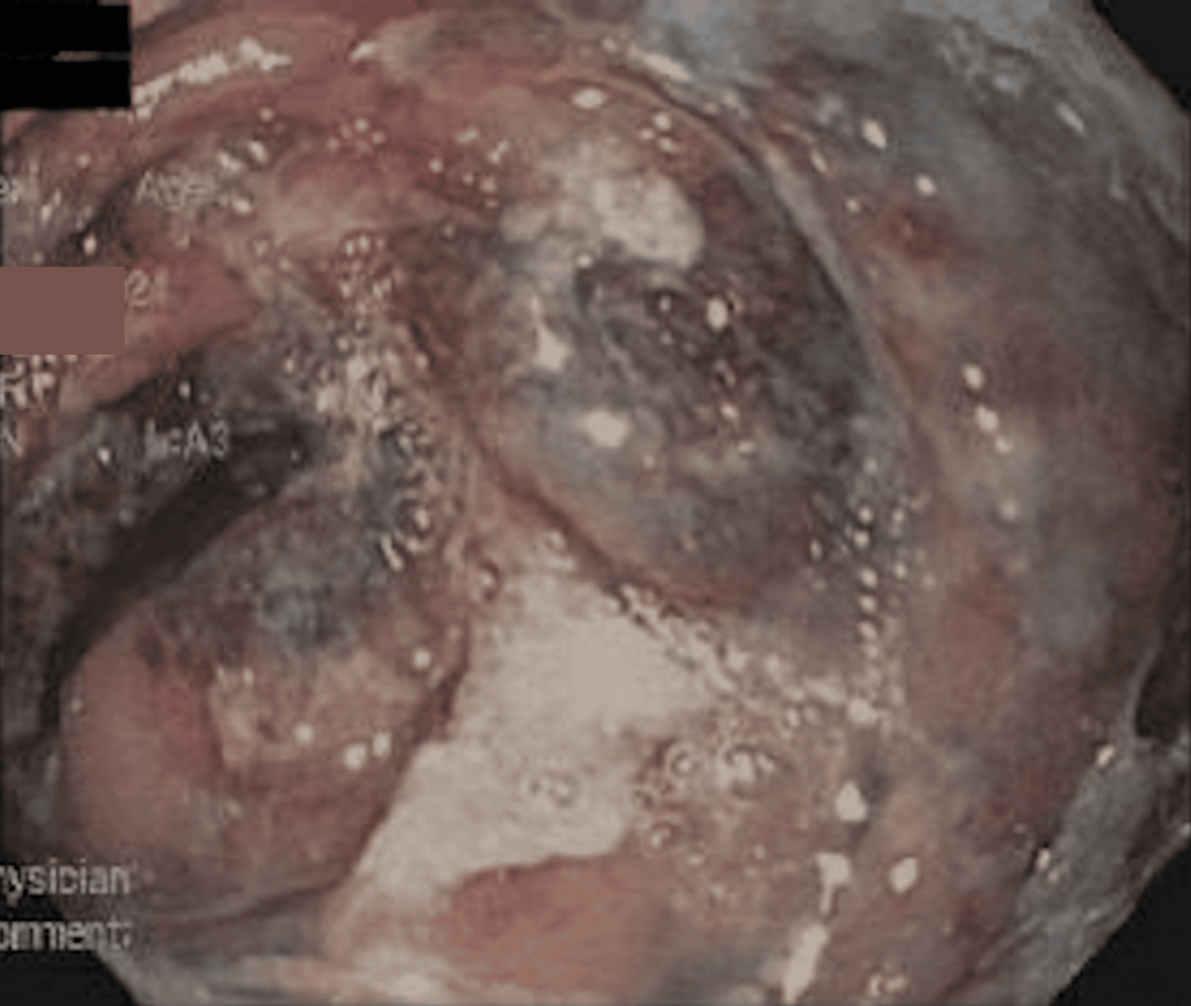
Endoscopic image of the jejunal limb (Roux limb) Diffuse abnormal mucosa with congestion, erythema, discoloration, and patchy necrosis with a hemorrhagic appearance.

Endoscopic evaluation revealed congestion, edema, erosions, and focal necrosis at the Roux-en-Y anastomosis (Figure 2). Moderate, patchy mucosal changes were observed in the cardia, gastric fundus, gastric body, and jejunum, characterized by congestion, discoloration, erythema, erosion, and a hemorrhagic appearance (Figures 3, 4). Additionally, focal necrosis was discovered in the jejunum. In response to the endoscopic findings, oral sucralfate was added for mucosal protection. The patient remained clinically stable following the above treatment plan with resolution of symptoms, leading to discharge from the hospital a few days later without the need for further surgical involvement.

## Discussion

Necrotizing gastroenteritis is a rare but severe condition characterized by intense inflammation and necrosis of the gastrointestinal mucosa, ultimately compromising bowel wall integrity and normal gastrointestinal function [13]. The pathophysiology remains incompletely understood, with both infectious and non-infectious mechanisms, as seen in our patient, contributing to its development.

Infectious causes include bacterial pathogens such as *Clostridium perfringens*, *Salmonella* spp., and* Escherichia coli*, along with viruses and fungi, and these can directly invade the mucosa or produce toxins that trigger inflammatory cascades [14]. These processes lead to vascular congestion, edema, and ischemia. Bacterial toxins further promote cell death and intestinal permeability, stimulating the release of cytokines and prostaglandins that exacerbate tissue damage [15].

In non-infectious causes, ischemia plays a central role. Reduced blood flow from causes such as hypoperfusion, shock, hypotension, vascular disease, vasculitis, or chronic mucosal injury can result in mucosal and submucosal necrosis [16]. Once mucosal integrity is lost, bacterial translocation and systemic infection may follow, worsening clinical outcomes.

Progressive necrosis leaves the bowel vulnerable to perforation, which can precipitate peritonitis and sepsis [17]. The resulting systemic inflammatory response contributes to electrolyte derangements, metabolic instability, and fluid shifts. As was seen in our patient, labs performed at the time of admission revealed significant leukocytosis with relative neutrophilia and lymphocytosis, mild microcytic anemia, and elevated red cell distribution width (Table 1). These complications account for the high morbidity and mortality associated with necrotizing gastroenteritis [17].

While most reports of hydrogen peroxide ingestion involve concentrated (35-50%) solutions [2,18], this case highlights that even 3% household formulations can cause significant gastrointestinal injury. Documented outcomes of several adult cases reinforce the various, but potentially severe, outcomes of hydrogen peroxide ingestion and the variation in management techniques. This includes the case of a 39-year-old man who ingested ~250 mL of 35% hydrogen peroxide, who subsequently developed portal venous gas and diffuse gastric injury, but recovered with conservative therapy [2]. Another case highlighted a 77-year-old man whose hydrogen peroxide ingestion was complicated by gastric pneumatosis, which was managed nonoperatively [19]. In contrast, two cases that improved with hyperbaric oxygen (HBO) therapy include that of a 52-year-old man who developed portal venous gas and diffuse hemorrhagic gastritis after ingestion of 35% hydrogen peroxide and a 30-year-old woman whose hydrogen peroxide ingestion was complicated by pneumatosis and pneumobilia [18,20]. 

Treatment remains individualized and depends on the severity of mucosal injury. Stable patients without evidence of perforation or full-thickness necrosis are often managed conservatively with bowel rest, intravenous proton pump inhibitors, and mucosal protectants [17]. HBO therapy has shown promise, particularly in cases complicated by portal venous gas, pneumatosis, or extensive ischemia, though access is limited [18,20].

In our patient, susceptibility to injury was likely increased by pre-existing gastric pathology and surgically altered anatomy, which predisposed the mucosa to necrosis as a result of decreased blood supply, stasis, and modified motility that prolonged exposure to gastric acid [21]. In addition, chronic reflux with a hiatal hernia and Barrett’s features, daily smoking, use of acid-suppressive medications, anemia with reduced oxygen delivery, and occasional alcohol intake likely increased the risk. Early presentation, detection, and treatment may have contributed to fewer abnormal lab results. This underscores the importance of early endoscopic evaluation to provide direct visualization of the mucosa, as radiologic imaging alone may underestimate the degree of mucosal injury. For this reason, endoscopy remains the gold standard for assessing caustic injury severity [2,20,21].

## Conclusions

This case demonstrates that caustic injury from even minute amounts of low-concentrated hydrogen peroxide can produce clinically significant mucosal necrosis and hemorrhagic gastritis in an otherwise healthy adult, a risk that is amplified in magnification through alteration of foregut anatomy following a history of Roux-en-Y gastric bypass surgery. Early recognition with prompt endoscopic evaluation remains the forefront method of evaluation, both to define the extent of mucosal injury and to guide management decisions; whereas, clinically stable patients without evidence of full-thickness necrosis can be successfully treated conservatively with bowel rest, proton-pump inhibitors, broad-spectrum antibiotics, and mucosal protective stimulating agents as indicated in this case.

In cases involving complications, such as portal venous gas, pneumatosis, or progressive ischemia, there may be a need for adjunctive therapies such as hyperbaric oxygen therapy or surgical intervention. This report underscores the need for clinician awareness of the potential hazards of household chemical ingestion, continual patient education and counseling after foregut surgery, and continual study to define prognostic indicators and standardized management pathways for caustic-induced necrotizing gastroenteritis. 
